# Global Research Trends in Community-Based Strategies for Reducing Risky Alcohol Consumption and Promoting Health

**DOI:** 10.3390/ijerph23010086

**Published:** 2026-01-08

**Authors:** Kristijan Breznik, Andreja Hrovat Bukovšek, Tamara Štemberger Kolnik

**Affiliations:** 1 Faculty of Health Sciences in Celje, 3000 Celje, Slovenia; andreja.hrovat-bukovsek@fzvce.si (A.H.B.); tamara.stemberger-kolnik@fzvce.si (T.Š.K.); 2 International School for Social and Business Studies, 3000 Celje, Slovenia

**Keywords:** community-based strategies, risky alcohol consumption, public health prevention, bibliometric analysis, global and local approaches, health policy, population health

## Abstract

**Highlights:**

**Public health relevance—How does this work relate to a public health issue?**

- Hazardous alcohol use is a major global risk factor, and this study maps how community-based strategies to reduce risky drinking have developed across countries, populations, and settings.
- By positioning alcohol-focused programs within broader health-promotion and prevention domains, the study shows how community interventions are embedded in mainstream public health practice.

**Public health significance—Why is this work of significance to public health?**

- The analysis identifies core thematic areas, especially youth and maternal and child health platforms, that currently drive community-based alcohol drinking prevention and are critical targets for public health action.
- It documents a shift from high-income dominance toward increasing contributions from low- and middle-income countries, highlighting both growing capacity and persistent inequities in alcohol-prevention research.

**Public health implications—What are the key implications or messages for practitioners, policy makers and/or researchers in public health?**

- Practitioners and policymakers can use these findings to prioritize community-based alcohol drinking prevention strategies that align with established health promotion, primary prevention, and maternal and child health frameworks.
- Researchers are directed toward understudied regions, populations, and delivery models, supporting more equitable, evidence-informed alcohol drinking policies and stronger integration of evaluation data into public health monitoring.

**Abstract:**

The aim of this study was to map global research on community-based strategies to reduce risky alcohol consumption and promote health, aiming to clarify growth, leading contributors, thematic structure, and integration with public-health frameworks. Using a PubMed corpus, we analyzed production, authorship, and collaboration indicators, built a thematic map (centrality/density) to identify core topics, and applied Multiple Correspondence Analysis to assess conceptual proximity between alcohol-specific and broader prevention domains. The dataset comprised 2607 documents across 916 sources, with output led by the USA, with substantial contributions from Australia, Canada, the UK, and rising activity in sub-Saharan Africa. The thematic map showed a mature core centered on adolescents and pregnancy, cross-cutting foundations in health education and sexual behavior with substance-related disorders, measurement-oriented niches at the periphery, and emerging work linking family planning. The Multiple Correspondence Analysis positioned alcohol-prevention terms close to health promotion, primary prevention, and epidemiology, with maternal–child health bridging community programs and clinical prevention. Overall, community-based alcohol prevention is expanding, globally distributed, and embedded in mainstream public-health practice. Limitations include the absence of citation data in PubMed, and future work should integrate citation-enabled databases and compare patterns across income groups.

## 1. Introduction

Hazardous alcohol consumption remains one of the most pressing global public health challenges, despite decades of professional and policy initiatives aimed at its reduction. According to the World Health Organization [1], the harmful use of alcohol is responsible for approximately 2.6 million deaths each year, accounting for a substantial share of the global burden of disease and mortality. Along with tobacco products and other psychoactive substances, alcohol represents one of the key determinants contributing to the loss of healthy life years worldwide [2]. Among the working-age population, the impact of alcohol consumption surpasses that of most other risk factors affecting both health and productivity [1,3]. Recent global bibliometric analyses further demonstrate a marked increase in research on harmful alcohol use, alongside a notable shift in focus from social determinants to biological mechanisms and intervention strategies [4]. Anderson et al. [5] emphasize that there is no safe level of alcohol consumption that can be associated with positive health outcomes. This view is supported by recent national guidelines, including the Canadian ones, which recommend a maximum of two drinks per week due to the linear increase in mortality risk even at low levels of alcohol intake [6,7]. In contrast, alcohol consumption, regardless of quantity, always entails a certain level of risk. Alarmingly, the adults who engage in risky drinking incorrectly believe that moderate alcohol use reduces the likelihood of developing chronic non-communicable diseases [5]. In Canada, for example, 77% of adults perceive consuming more than two drinks per week as posing little or no risk [7].

Alcohol often plays an important social and cultural role, particularly as part of leisure and tourism activities [8]. Bibliometric analyses reveal that most research on alcohol focuses on the determinants and social contexts of drinking, with the majority of studies originating from high-income countries [9]. The issue of risky drinking is also prominent among young people. Data from the European School Survey Project on Alcohol and Other Drugs (ESPAD) indicate that while the overall prevalence of alcohol use and intoxication has been declining, the decrease is more pronounced among boys, whereas it remains relatively modest among girls. Across many countries participating in the survey, alcohol remains widely available, with early drinking initiation and episodic heavy drinking continuing to cause substantial health and social consequences [10].

Global mapping of the literature on underage drinking suggests an increasing focus on environmental and community-based measures aimed at reducing alcohol availability, with regulatory approaches being the most common and effective [11]. Tschorn et al. [12] found a statistically significant association between social and personality factors and alcohol misuse among adolescents. Their findings suggest that risky drinking often develops as a result of the brain’s reward system functioning, while family patterns and individual risk factors also play a crucial role. Similarly, Jager et al. [9] highlight the influence of family environment, peer relationships, and local alcohol availability on the development of early drinking patterns. Such early behavioral patterns may persist into adulthood and lead to chronic alcohol dependence. Among young people, hazardous alcohol consumption is even one of the leading causes of mortality [1].

In low- and middle-income countries, harmful drinking among youth is rising rapidly, as adolescents increasingly become a target of the alcohol industry’s marketing strategies [13]. The risk is particularly pronounced during adolescence, as behavioral patterns formed early in development may have long-lasting adverse consequences. Longitudinal data from the Add Health study confirm that protective factors within the family, school, and local community substantially reduce the likelihood of harmful drinking and other risky behaviors among adolescents [14]. Studies from Africa further confirm that access to community-based prevention programs plays a key protective role among young people [15]. Globally, alcohol ranks as the sixth leading risk factor for premature mortality and morbidity [1], underscoring the need for targeted programs to reduce harmful drinking, particularly among vulnerable population groups. Legislative changes introducing zero blood alcohol concentration limits have reduced traffic accidents in Brazil and Uruguay [16,17], while the most successful initiatives are community-based programs combining social mobilization, media campaigns, and consistent law enforcement [18].

Nilsson et al. [19] emphasize that local initiatives can play a crucial role in reducing the harmful consequences of risky alcohol consumption. Despite the proven effectiveness of community-based prevention programs. Ure et al. [20] caution that such measures should not rely solely on voluntary engagement but require stable financial support and a clear national policy framework aimed at reducing risky drinking across all population groups. Similarly, Reynolds et al. [21] highlight the importance of linking national and local decision-making levels, as this facilitates more effective legislative development, the creation of new policy instruments, the inclusion of diverse experts in strategy formulation, and continuous stakeholder engagement in policy design.

In addition to preventive and educational measures, economic policies exert a significant influence. Economic research [22] examining the effect of the full price of alcoholic beverages on consumption has shown that price increases, implemented by national or local policymakers, can delay the initiation of drinking among adolescents and reduce overall population-level alcohol consumption. Measures such as higher excise taxes and minimum pricing have been shown to mitigate harmful outcomes, including alcohol misuse, drink-driving, traffic accidents, violence, and other criminal offenses [23]. Evidence from meta-analyses confirms that taxation, minimum unit pricing, and sales restrictions reduce alcohol consumption by 3–10% and prevent hospitalizations and deaths [24]. The introduction of a minimum unit price for alcohol has proven particularly effective, reducing alcohol-attributable mortality in Scotland by 13.4% [25]. Higher prices for alcoholic beverages, therefore, demonstrably decrease total alcohol consumption and contribute to long-term public health improvement [26].

Berhe et al. [27], in a meta-analysis of studies evaluating the effectiveness of interventions to prevent risky alcohol consumption, found that participants from low- and middle-income countries significantly reduced hazardous drinking following intervention programs. In contrast, studies from high-income countries did not report significant differences in alcohol use between intervention and control groups.

Research further indicates that successful local prevention efforts to reduce risky alcohol consumption can be effectively integrated with global strategies through transferable models and adaptable approaches. The preventive pilot project in Poland [28] achieved a substantial reduction in risky drinking through a multicomponent community-based approach. The model was built upon the establishment of multidisciplinary local teams (including social workers, psychologists, and addiction therapists), tailored interventions based on risk levels, and intersectoral collaboration between healthcare, social services, and non-governmental organizations. The long-term effectiveness of local programs is also supported by the preventive program in Stockholm [29], which has maintained a 76.7% rate of refusal to serve alcohol to intoxicated patrons over two decades.

Key factors contributing to success and global transferability include program institutionalization, continuous staff training, systematic monitoring and evaluation, and adaptation to local sociocultural contexts. In a systematic review, Sánchez-Puertas et al. [30] identified four core areas of preventive action with potential for global implementation: (i) school-based programs that effectively change attitudes and reduce acceptance of alcohol among youth; (ii) family-based programs that strengthen communication and parenting skills; (iii) community-based programs that foster protective environments; and (iv) digital interventions that ensure wide reach and accessibility.

Jalloh et al. [31] from the West African region further emphasize that non-governmental and community-based organizations play a pivotal role in prevention, but require strengthened institutional capacities and access to relevant information. This underscores the need for international knowledge exchange, standardized training, and adaptation of global strategies to local contexts. Evidence supporting the effectiveness of community-based strategies for reducing risky alcohol consumption derives from a wide range of research sources, among which bibliometric analyses are gaining increasing importance. These analyses enable systematic monitoring and evaluation of research trends at the global level. Zyoud [4] conducted a comprehensive bibliometric analysis of studies on so-called binge drinking, encompassing 2763 scientific articles from 139 countries. The findings illustrated how research trends in this area have evolved over time and identified the countries and research groups that have made the most substantial contributions to scientific development. According to the analysis, publications on binge drinking peaked around 2018, with the United States accounting for 56.1% of all outputs.

Ninkov et al. [32] describe bibliometrics as a research method analogous to epidemiology, as it utilizes data on scientific publications rather than patient data to understand research “populations.” This approach provides valuable insights into the evolution of scientific fields, the identification of leading researchers, institutions, and regions, and the mapping of thematic shifts in research focus. Mejía et al. [33] further highlight the role of bibliometrics in public health, emphasizing its utility in science mapping, tracking research trends, and supporting evidence-informed policymaking. By combining quantitative publication data with thematic analysis, bibliometrics offers a unique perspective on how scientific research responds to social challenges, including risky alcohol consumption. This contributes to a broader understanding that global research orientations and local preventive practices are not separate phenomena but interconnected processes. While bibliometric analyses uncover structural trends and gaps within the research landscape, local community-based interventions, such as those demonstrated in successful practice models [28,29,30], provide empirical foundations for developing evidence-based, transferable approaches.

Overall, global research trends and local preventive initiatives appear to be complementary: global analyses identify effective mechanisms and directions, whereas local practices offer concrete models that can be transferred, adapted, and integrated into a broader global framework of public health interventions. Existing bibliometric studies on alcohol research tend to emphasize overall alcohol use, treatment, or policy effects, but rarely isolate community-based prevention as a distinct domain or examine how it integrates with broader public-health frameworks. They also provide limited coverage of low- and middle-income countries contributors and seldom quantify conceptual linkages between alcohol-specific terms and general prevention concepts. To address this gap, we present a content-focused mapping of community-level alcohol-risk prevention using thematic mapping (centrality and density) and Multiple Correspondence Analysis, with explicit tests of linkage strength and attention to geographic and institutional distribution, thereby revealing the field’s core engines and degree of alignment with mainstream public-health practice. We postulated the following research questions:

RQ1: How has research output on community-based strategies for reducing risky alcohol use evolved over time?
RQ2: Which countries and institutions are most active in this field?
RQ3: What thematic dimensions structure the research content on community-based strategies to reduce risky alcohol consumption and promote health?
RQ4: How strongly is alcohol prevention linked to broader prevention concepts?

## 2. Materials and Methods

We searched PubMed (MEDLINE) to identify studies on community-based strategies for reducing risky alcohol consumption within health promotion and prevention. We structured the PubMed search around three concept blocks: (1) Community/local/settings: terms capturing community-based or local-level approaches (e.g., community health services, community interventions, healthy/local communities) to exclude purely clinical or hospital settings unless explicitly community-linked; (2) Alcohol risky drinking: subject headings and free-text terms focused on alcohol use and its risky and harmful patterns (including prevention and harm reduction) to ensure alcohol-specific retrieval and avoid single-substance studies unrelated to alcohol; (3) Health promotion and prevention: headings and terms anchoring the preventive public-health framing (health promotion, primary prevention, risk reduction, public health) to filter out treatment-only literature. Within each block, we combined synonyms with OR (including truncation to capture word variants) and intersected the three blocks with AND. Furthermore, the search combined Medical Subject Headings ([MeSH]) and free-text terms in titles/abstracts ([tiab]), using Boolean operators and truncation. The search strategy was divided into three core conceptual blocks. The final query was:

(“Community Health Services”[Mesh] OR “Community Health”[tiab] OR “community-based”[tiab] OR “community intervention*”[tiab] OR “local communit*”[tiab] OR “healthy communit*”[tiab])

AND;

(“Alcohol Drinking/prevention and control”[Mesh] OR “Alcohol-Related Disorders/prevention and control”[Mesh] OR alcohol[tiab] OR “alcohol use”[tiab] OR “alcohol consumption”[tiab] OR “risky drinking”[tiab] OR “hazardous drinking”[tiab] OR “harmful drinking”[tiab] OR “alcohol prevention”[tiab] OR “alcohol harm reduction”[tiab])

AND;

(“Health Promotion”[Mesh] OR “Primary Prevention”[Mesh] OR “health promotion”[tiab] OR prevention[tiab] OR “risk reduction”[tiab] OR “public health”[tiab]).

On the obtained dataset, we conducted a bibliometric and science-mapping analysis in order to quantify research production and to characterize the intellectual structure of the field. Descriptive indicators included annual output, sources, authorship, and international collaboration. For science-mapping, we used keyword-based thematic mapping and conceptual structure via Multiple Correspondence Analysis to examine thematic organization and its linkage to broader prevention domains. All analyses were performed in R [34] with the bibliometrix package [35], using its standard workflow and functions for production indicators, collaboration metrics, thematic mapping, and the MCA. Because the dataset derives from PubMed, which omits cited-reference metadata, citation-based indicators (co-citation, bibliographic coupling) were not computed here. Results rather focus on content and keyword structure, productivity and collaboration metrics.

Multiple Correspondence Analysis (MCA) is an exploratory method for summarizing patterns in categorical data [36]. In our case, the presence or absence of keywords across documents is analysed. MCA places terms (and optionally documents or term groups) in a low-dimensional map where the distance between points reflects how often they co-occur. Items that appear together more frequently are positioned closer, while rarely associated items are positioned farther apart. The first one or two dimensions in the MCA capture the most salient contrasts in the data, giving an intuitive “conceptual space” of the field.

MCA is well-suited to bibliometric applications because it does not assume linear relationships or numeric scales. Instead, it uses the co-occurrence structure of descriptors (in our case, author keywords) to reveal themes, oppositions, and bridges among topics. It has been widely used in a similar context to support bibliometric results [37]. Interpreting an MCA map involves reading broad axes (what differentiates the left–right and up–down directions), noting clusters of related terms, and judging proximity between focal domains. Closer proximity suggests stronger conceptual integration.

## 3. Results

The final dataset comprises 2607 documents published across 916 sources over the period 1954–2025 (the last year not finished yet), with an annual growth rate of approximately 6.9% and a mean document age of 13.8 years. This indicates a long historical tail and sustained recent expansion. Authorship is relatively broad; 12,997 unique authors contributed, with a typical paper consisting of 6.2 co-authors. Regarding authorship, 253 of the papers were single-authored, and approximately 10.2% involving international co-authorship, which points to moderate cross-border collaboration. Topic diversity is reflected in the vocabulary, based on keywords, 5125 distinct terms were found.

The remaining part of this section is divided into four subsections that present complementary analyses describing the scope and structure of research on community-based strategies to reduce risky alcohol consumption and promote health. First, Temporal Trends in the Literature (related to RQ1) summarizes annual scientific production and topic dynamics over time. Second, Geographic and Institutional Contributions (RQ2) profiles the most active countries and leading affiliations, highlighting collaboration patterns. Third, Thematic Structure of Community-Based Alcohol Prevention (RQ3) maps the field’s core themes using a thematic map. Finally, Integration with Public Health Frameworks (RQ4) examines how alcohol prevention links to broader prevention and health-promotion concepts using conceptual MCA analysis.

### 3.1. Temporal Trends in the Literature

This subsection frames the field historically by tracing how research activity has unfolded over time and how its emphases have shifted. We first chart annual scientific production to show the pace and continuity of work, then use a trend-topics view to outline the main trajectories in focus and methodology.

**Figure 1:**
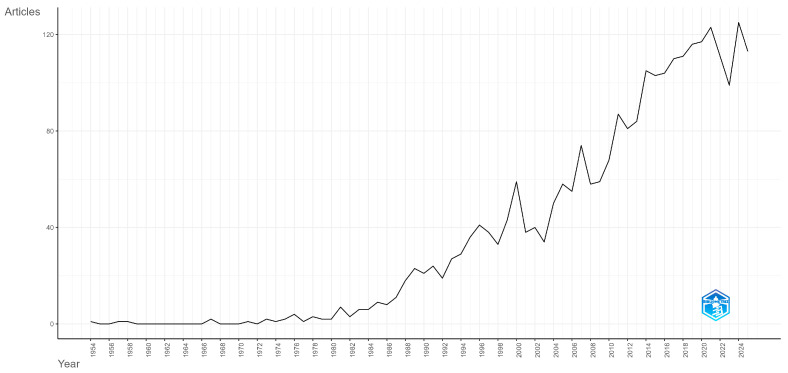
Annual scientific production of documents on the topic of reducing risky alcohol consumption and promoting health.

Analyzing Figure 1, documents were sparse until the late 1980s, followed by a steady rise through the 1990s and early 2000s. Output accelerated sharply after the year 2010, reaching over 100 articles per year in the early–mid 2020s with year-to-year volatility (short dips followed by new highs). In general, the field shows sustained growth of documents over three decades, with its highest volumes in the most recent years.

**Figure 2:**
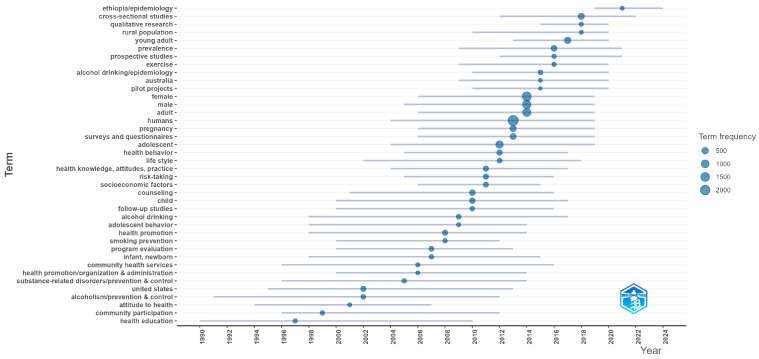
Trend topics over time with frequencies.

Trend topic analysis over time is presented in Figure 2 and provides the evolution of topics through the analyzed period.

Early works shown in Figure 2 emphasized health education, community participation, and broad health promotion with organization and administration themes. Through the 2000s, topics concentrated on alcoholism prevention and control, community health services, and program evaluation, alongside youth-oriented terms (adolescent behavior, school with a child focus). From 2010 onward, the vocabulary broadened and methods diversified: frequent terms include surveys and questionnaires, health behavior, risk-taking, follow-up studies, and counseling; design tags such as cross-sectional, prospective, and qualitative research became prominent. Recent years show more epidemiology and prevalence labels, population tags (young adult, female and male, pregnancy), contextual terms (rural population), and geographic markers (e.g., Australia, Ethiopia), indicating wider settings and regions.

### 3.2. Geographic and Institutional Contributions

This subsection identifies countries and institutions that drive the field and where research capacity is concentrated. Country and affiliation rankings highlight the main hubs of expertise, the extent of international collaboration, and potential imbalances that shape what gets studied and funded. Reading these lists alongside collaboration patterns helps identify emerging centers, policy-relevant networks, and opportunities to broaden participation. In Table 1, a list of the top 25 countries regarding document production is shown, followed by Table 2 with the top 25 institutions.

The United States dominates the field with 734 publications (just over 28% of total output). With other Anglophone countries, such as Australia (5.4%) and Canada (4%), form a strong group, consistent with well-established community-based public health infrastructures and prevention funding mechanisms. Notably, Ethiopia (3.2%) ranks surprisingly high, which highlights the growing contribution of sub-Saharan Africa to alcohol and health promotion research.

Overall, the regional pattern shows a transition from Western dominance toward a more globally distributed research landscape. High-income countries still account for the majority of studies, but the increasing representation of Africa, Asia, and the Global South suggests a broadening recognition of community-level alcohol risks and preventive strategies in diverse social and economic settings. The moderate rates of international collaboration (MCP% values of 6–12%) point to emerging global research networks, especially between high- and middle-income regions, though collaboration intensity remains lower than in some other public health domains.

The affiliation landscape is dominated by large, research-intensive Anglophone universities, most of which also appear among the top institutions worldwide in the 2025 QS World University Rankings for Life Sciences & Medicine [38]. The University of California system, ranked first by publication volume in this dataset and positioned within the global top tier in QS rankings, exemplifies the depth of U.S. capacity in community health and alcohol-prevention research. The University of Louisville, though a relatively smaller player internationally, contributes a distinctive and consistent programmatic line in community-based prevention, explaining its high publication count relative to size. Other leading U.S. institutions—including the University of Washington, Columbia University, and Johns Hopkins Bloomberg School of Public Health—reinforce the United States’ well-documented leadership in public health and alcohol-related behavioral research.

In Canada, the University of Toronto (a QS top-20 institution globally in life sciences and medicine) and McMaster University serve as national hubs of public-health scholarship, emphasizing evidence-based health promotion and community health interventions. The United Kingdom contributes several globally recognized centers, notably the London School of Hygiene and Tropical Medicine and King’s College London, both ranked within the top 50 worldwide for life sciences and medicine. These universities anchor a strong European public-health research tradition and act as central nodes in international consortia.

A particularly visible Australian cluster—comprising Monash University, the University of Newcastle, Deakin University, and the University of New South Wales—reflects that country’s sustained national investment in health promotion infrastructure, school-based prevention, and alcohol harm reduction programs. All four institutions hold high QS positions (Monash and UNSW among the top 50 globally in life sciences and medicine), underscoring both research excellence and practical engagement with community health policies.

Several sub-Saharan African institutions stand out, the University of Gondar and Haramaya University (both in Ethiopia) and the University of Cape Town (South Africa, a QS top-100 life sciences university) contribute a growing share of the literature. Their prominence signals a tangible diffusion of research capacity and leadership toward low- and middle-income settings, consistent with the broader geographic diversification evident in the dataset and the rising engagement of African universities in WHO-aligned community health initiatives.

Beyond individual universities, several of the most productive institutions identified in this study are embedded within broader institutional networks that shape alcohol prevention research and policy. These include WHO Collaborating Centres in public health and substance use, national public health institutes, and large university-based schools of public health that act as hubs for international collaboration and evidence translation [1,9,13]. Institutions such as the London School of Hygiene & Tropical Medicine, Johns Hopkins University, and the University of Toronto are closely linked to national and global policy processes, contributing research evidence that informs WHO strategies, national alcohol control policies, and community-based implementation frameworks [1,9,21]. Through their involvement in guideline development, surveillance systems, and policy advisory roles, these institutions help bridge scientific production and policy action at both global and country levels.

### 3.3. Thematic Structure of Community-Based Alcohol Prevention

This subsection profiles the field’s conceptual structure using a thematic map. Clusters of keywords are positioned by centrality (how strongly they connect to the rest of the field) and density (how internally developed they are), yielding four intuitive quadrants: motor (central, well-developed), basic (central, broad foundations), niche (specialized, less connected), and emerging/declining (peripheral, less developed). Reading the map, we explore which themes currently drive community-based alcohol prevention, which provide cross-cutting foundations, and which represent specialized or nascent lines of work.

**Figure 3:**
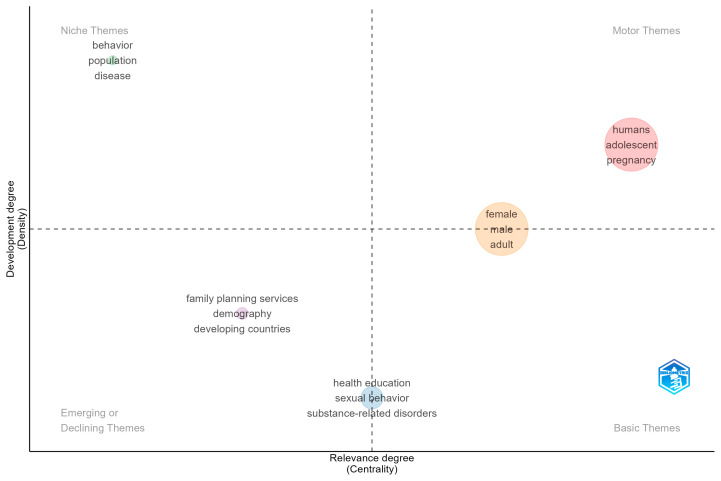
Thematic map of Community-Based Alcohol Prevention.

The thematic map in Figure 3 shows two highly central clusters that organise the field. The largest, more developed cluster groups adolescents and pregnancy, indicating that youth-oriented prevention and maternal contexts form the main engines of community alcohol-risk work. A second, moderately developed but still central cluster consists of female, male, and adult, reflecting routine sex- and age-stratified analyses in general-population studies that connect broadly across topics, albeit with less internal cohesion than the adolescent–pregnancy stream. At the base of the map, we can find health education, sexual behavior, and substance-related disorders as basic themes. They are widely connected foundations of community prevention, but conceptually broad and therefore lower in internal density. In the niche quadrant, behavior, population, and disease appear as specialised areas that are well developed internally yet less connected to the core prevention discourse. The emerging/declining quadrant contains family planning services, demography, and developing countries, suggesting a growing, though still peripheral, line of work that links alcohol-risk prevention to reproductive-health platforms and to programmes in low- and middle-income settings.

Taken together, the map indicates that community alcohol-risk prevention is prevailing in youth and maternal health platforms, while adult and sex-stratified work remains broad and heterogeneous. Health education functions as a cross-cutting connector rather than a tightly bound specialty, consistent with settings-based health promotion that co-targets sexual behaviour and other risk factors alongside alcohol. Specialised behavioural and population methodologies contribute measurement depth without yet shaping the field’s spine. The presence of family planning and low- and middle-income country-oriented terms in the emerging quadrant aligns with the geographic diversification, indicating a likely growth frontier for implementation research and policy integration in primary care and reproductive-health services.

By interpreting Figure 3, centrality reflects how strongly a theme is connected to other themes (field relevance), while density reflects its internal development. The size of a bubble corresponds to the theme frequency. Generic indexing terms (e.g., humans) sometimes co-locate with substantive labels, as here they should be read as metadata artifacts that enlarge but do not define the adolescent–pregnancy cluster. Overall, the quadrant positions provide a concise roadmap where the field’s motor themes are youth/maternal platforms, basic themes anchor broad prevention practice, niche themes house method-heavy subfields, and emerging themes signal where new integration and regional expansion are most likely to occur.

### 3.4. Integration with Public Health Frameworks

**Figure 4:**
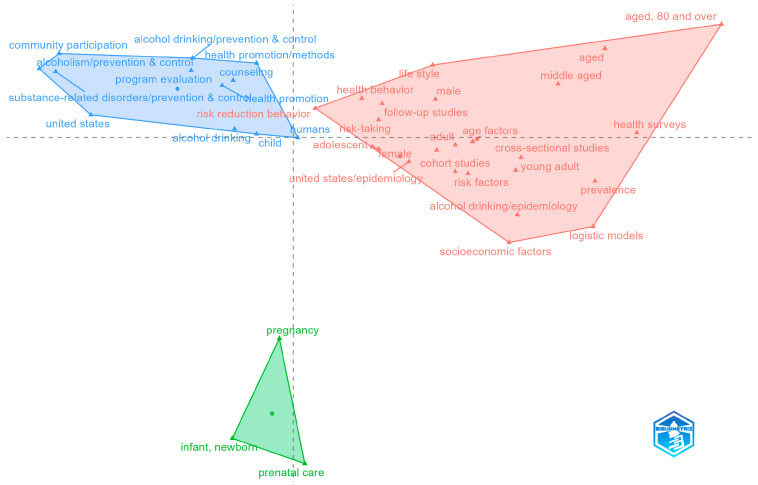
Conceptual space of community alcohol prevention: an MCA of thematic linkages.

This subsection examines how research on community-based alcohol prevention integrates with broader public-health frameworks. Using the MCA, we project keywords into a shared conceptual space. Assess proximity between alcohol-specific terms with prevention and health-promotion domains, where closer positions indicate stronger linkage. In Figure 4, we report the main clusters and relative distances to show where alcohol prevention aligns with programmatic health promotion, population epidemiology, and life-course services, providing a concise view of conceptual integration.

The conceptual space in Figure 4 resolves into three coherent clusters that together show how community alcohol-risk prevention is embedded in mainstream public-health practice. On the left, a programmatic health-promotion and prevention cluster (community participation; alcohol drinking: prevention with control; health promotion and methods; counseling; program evaluation; risk-reduction behavior) anchors the settings-based approach. This is familiar from the Ottawa Charter, which includes communities, education, and organized prevention programs [39]. The presence of “United States” and “child” within this space reflects large bodies of U.S. community programming and schools with child-focused initiatives. On the right, an epidemiology and measurement cluster (health surveys; prevalence; risk factors; cross-sectional and cohort studies; logistic models; socioeconomic factors; age factors with young adult/middle-aged/aged, including ≥80) indicates strong integration with population-health surveillance frameworks. This pole captures the quantification of alcohol-related risks across the life course and by social determinants, linking community prevention to monitoring systems and evidence standards typical of public-health epidemiology. At the bottom, a focused maternal–child health cluster (pregnancy; prenatal care; infant and newborn) signals the use of maternal–child health platforms for alcohol-risk screening, counseling, and prevention. This is an integration point between community services and primary care with reproductive health.

Taken together, the results of the MCA suggest that research in this field is structured around two complementary pillars: the first on settings-based health promotion and the second population-level epidemiology. The third identified pillar shows the area of maternal and child health and represents the connection between the first and second pillars, drawing attention to a population that is particularly susceptible to improving self-efficacy in health behaviour. The health-promotion side delivers community participation, counseling, and evaluation. The epidemiologic side adds risk-factor surveillance and an equity lens (e.g., socioeconomic factors). The maternal–child health node provides the clinical–public-health bridge for targeted prevention during pregnancy and early life.

Spatial proximity in the MCA map indicates conceptual linkage; keywords plotted closer together co-occur more frequently across documents. The first (horizontal) axis contrasts health-promotion and program delivery on the left with epidemiology and measurement on the right, while the second (vertical) axis separates maternal–child health service platforms below from general population measurement above. Cluster polygons trace the convex hull of terms, and the central dot marks each cluster centroid. Generic indexing tags (e.g., humans, geographic labels) may co-locate with substantive terms. These should be read as metadata artifacts that do not alter the substantive interpretation.

## 4. Discussion

The bibliometric and conceptual analysis demonstrates that research on community-based strategies to reduce risky alcohol consumption has undergone a distinct process of maturation over the past three decades. The observed publication trajectory, with early sporadic studies before the 1990s, steady consolidation during the 2000s, and rapid expansion after 2010, confirms the emergence of this field as a coherent component of public health science. This sustained growth does not indicate saturation, but rather methodological consolidation and policy relevance, reflecting an increasing emphasis on complex, evidence-based community interventions [4,13].

The thematic evolution from generic notions of “community participation” and “health education” to design- and outcome-oriented frameworks parallels global trends in prevention research, where evaluation, effectiveness, and population specificity have become central [9]. This finding aligns with earlier studies indicating that community-based interventions are most successful when grounded in measurable outcomes and multilevel approaches integrating regulation, education, and mobilization [11,18]. Consistent with Berhe et al. [27], the present analysis underscores that long-term, multicomponent community programs—particularly those implemented for at least one year—yield the most reliable and sustained effects, supporting the need for institutional continuity and systematic evaluation capacity within community health systems. The shift from foundational community health and education keywords to methodological and evaluation-oriented terms suggests the field has moved from program initiation toward measurement, outcomes, and comparative designs. Increasing appearance of population tags (youth, young adults, pregnancy) and settings (rural) signals more granular targeting and equity-focused contexts beyond high-income urban studies. Growth in cross-sectional and prospective studies with qualitative labels reflects mixed-methods and epidemiologic expansion. This is consistent with larger, multisite, or implementation studies [40].

Geographically, the analysis confirms a persistent concentration of research within Anglophone academic systems, particularly the United States, Canada, the United Kingdom, and Australia. However, recent years reveal increasing leadership from low- and middle-income countries, marking a transition from passive data collection to active research co-development. This shift reflects the growing scientific capacity and political commitment observed in regions such as Africa, Asia, and Latin America. The findings resonate with Tapera et al. [15], who documented the rise of African-led alcohol-prevention research, and with Guimarães et al. [17], whose evidence from Brazil and Uruguay shows that locally embedded, community-based programs within national policy frameworks can significantly reduce alcohol-related harm. Similarly, interventions implemented in Ethiopia and Uganda [27] demonstrated measurable reductions in risky alcohol use when community engagement was combined with supportive public policies. The growing diversification of research output—both geographically and thematically—reflects a broader global movement toward inclusivity and context-sensitive prevention. Studies from Asia, such as those by Li et al. [41], confirm that school- and workplace-based interventions are most effective when aligned with broader health-reform strategies, echoing the priorities of the WHO Global Strategy to Reduce the Harmful Use of Alcohol. Collectively, these developments highlight the convergence between global research agendas and localized community practice, suggesting that effective alcohol prevention depends on the dynamic interaction between scientific evidence, local engagement, and policy integration. Importantly, the country-level patterns identified for RQ2 are closely mirrored by institutional concentration within a limited number of globally connected universities and public health institutes. These institutions function as knowledge brokers between research and policy, enabling countries with strong institutional infrastructures to translate community-based evidence into national prevention strategies more effectively than settings where such institutional networks are still emerging.

By region, publication output is concentrated in North America, led by the University of California (155), University of Louisville (140), University of Washington (73), Columbia (66), and Johns Hopkins (61). Canada contributes strongly via the University of Toronto (96), McMaster (52), and UBC (49). In the UK/Europe, the London School of Hygiene & Tropical Medicine (63), King’s College London (47), and UCL (41) anchor activity, while Australia forms a cohesive cluster—Monash (78), Newcastle (78), Deakin (73), and UNSW (47). Notably, sub-Saharan Africa adds substantial output from Gondar (94), Hamaya (58), and Cape Town (45), indicating growing leadership from LMIC settings. Many of these institutions also rank highly in the 2025 QS Life Sciences & Medicine lists (e.g., Johns Hopkins, UC, Toronto, UCL, Monash), suggesting alignment between productivity and broader research reputation. Conversely, several highly productive centres (e.g., Louisville, Gondar, Haramaya) are not QS-listed, underscoring the role of mission-driven prevention programs beyond global prestige metrics. Because our PubMed corpus does not include citation data, we emphasize publication counts.

Thematic and correspondence analyses in this study further elucidate the internal organization of the field. The stable conceptual core, anchored in youth, maternal, and primary prevention contexts, demonstrates that community-based alcohol prevention increasingly operates through established delivery interventions such as schools, maternal and child health services, and community health centres [30,42]. These interventions serve as key interfaces between public health institutions and populations, providing continuity across life-course stages. The strong conceptual association with health promotion, primary prevention, and population health measurement indicates the field’s deep integration into the foundational paradigms of contemporary public health. This interpretation is reinforced by recent systematic evidence. Oliveira et al. [11] and Sánchez-Puertas et al. [30] both identified schools, family settings, and community networks as the most effective and sustainable platforms for prevention—especially when supported by digital tools and policy-level actions. Similarly, Kilian et al. [43] and Nilsson et al. [19] emphasized that prevention is most effective when health-promotion principles are embedded within regulatory frameworks, allowing local programs to operate as extensions of national public health systems. These findings align with our bibliometric and conceptual results, confirming that the field has evolved from fragmented pilot initiatives into an integrated, evidence-based component of global prevention science.

The results of this study, therefore, contribute to extending current knowledge by demonstrating how bibliometric trends advocate for a systemic, multilevel approach to drinking alcohol prevention. They demonstrate that global research trends and local interventions are mutually reinforcing: bibliometric analyses reveal structural evolution and methodological innovation, while community-level evidence validates practical effectiveness and policy relevance. Together, these complementary dimensions illustrate that the science of community-based alcohol prevention has achieved conceptual and institutional maturity, positioning it as a vital pillar of modern public health.

The findings have clear implications for public health policy. The strong integration of community-based alcohol prevention with health promotion, primary prevention, and population surveillance indicates that such interventions are most effective when embedded within national public health systems rather than implemented as isolated projects [1,19,21,39]. Countries with strong research and institutional capacity demonstrate how sustained policy support and intersectoral coordination enable long-term community prevention, while for low- and middle-income countries the growing institutional participation highlights opportunities to scale prevention through alignment with primary health care and community health platforms, in line with WHO recommendations [1,13,30,31].

## 5. Conclusions

This bibliometric and conceptual analysis reveals that research on community-based strategies to reduce risky alcohol consumption has progressed through a clear developmental trajectory, from an initial formative phase to scientific consolidation and methodological maturity. The marked growth in publications after 2010 reflects the field’s establishment as an integral component of public health science, increasingly linked to policy development, prevention frameworks, and international research networks. Although research output remains concentrated in Anglophone countries, authorship and institutional participation are steadily diversifying toward the Global South, particularly in Africa, Asia, and Latin America. This shift signals the emergence of a more balanced and inclusive global research landscape, supported by expanding scientific infrastructure and stronger political commitment in low- and middle-income contexts. Thematic and correspondence analyses identified a stable conceptual core centered on youth, maternal, and primary prevention contexts. Key delivery interventions that bridge community-based practice and institutional public health systems. Their integration with health promotion and population risk measurement illustrates a life-course, systems-oriented framework that defines a mature, policy-relevant research domain.

Future work should reinforce longitudinal and comparative approaches and strengthen the integration of evaluation data into public health monitoring, ensuring equitable, evidence-based prevention strategies worldwide. Collaborative global networks and open access data sharing could further enhance cross-country comparability and accelerate evidence translation into policy. Sustained development of this research domain will be essential for ensuring equitable access to effective, evidence-based community strategies to reduce risky alcohol consumption worldwide.

Regarding the methods, we restricted the corpus to PubMed (MEDLINE) to leverage MeSH-based retrieval and ensure a uniform indexing scheme for thematic mapping and MCA. This choice prioritizes reproducibility and conceptual clarity within public-health practice. Our work can be extended beyond PubMed by linking the current corpus (via PMIDs/DOIs) to citation-enabled sources such as Scopus, Web of Science, or OpenAlex. This would allow full cited-reference lists, field-normalized citation indicators, co-citation and bibliographic-coupling analyses, and clearer identification of seminal works versus current research fronts. With richer metadata, it is possible to contrast high-income and low- and middle-income subcorpora directly. Collaborative global networks and open access data sharing could further enhance cross-country comparability and accelerate evidence translation into policy.

On the content side, complementing the thematic map with a co-word network and social network analysis would be useful. Collaboration networks could be examined for brokerage and bridging roles to see which institutions connect high-income and low- and middle-income communities, and whether equity-relevant topics (e.g., rural settings, socioeconomic gradients) cluster differently across income groups.

Also, temporal modeling, trend topics by quinquennium, and dynamic topic models can provide further results. Policy linkage can be tested with interrupted time-series around major guidance releases (Ottawa-inspired charters, WHO action plans), asking whether topic mix or network connectivity shifts after those milestones. Finally, emphasize robustness and reproducibility: normalize author/affiliation/funder names, report both full and fractional counting, vary keyword frequency thresholds, and vocabularies would be helpful.

### Limitations

This study has some limitations that should be considered when interpreting the findings. First, the analysis was based exclusively on PubMed, which does not provide cited-reference data, limiting the use of citation-based indicators such as co-citation or bibliographic coupling and constraining the identification of seminal works versus emerging research fronts. Second, institutional affiliations were analyzed as listed in PubMed records, which may underrepresent contributions from multi-institutional consortia, governmental agencies, or non-academic actors involved in community-based prevention. Third, publication counts reflect research activity rather than policy implementation or real-world impact, and thus cannot be interpreted as direct measures of effectiveness. Finally, although the study highlights geographic diversification, it does not disaggregate findings systematically by income group or policy regime, which should be addressed in future comparative analyses.

## Figures and Tables

**Table 1:** List of 25 most productive countries.

Country	Articles	Articles %	SCP ^1^	MCP ^2^	MCP %
USA	734	28.2	690	44	6.0
Australia	141	5.4	125	16	11.3
Canada	103	4.0	91	12	11.7
Ethiopia	83	3.2	75	8	9.6
India	68	2.6	57	11	16.2
China	58	2.2	45	13	22.4
Sweden	48	1.8	44	4	8.3
South Africa	46	1.8	31	15	32.6
Netherlands	39	1.5	32	7	17.9
Korea	36	1.4	33	3	8.3
United Kingdom	24	0.9	18	6	25.0
Japan	22	0.8	19	3	13.6
Nigeria	19	0.7	13	6	31.6
Spain	19	0.7	16	3	15.8
Brazil	17	0.7	14	3	17.6
Germany	16	0.6	11	5	31.3
New Zealand	16	0.6	12	4	25.0
Uganda	15	0.6	9	6	40.0
France	14	0.5	12	2	14.3
Thailand	13	0.5	10	3	23.1
Denmark	12	0.5	6	6	50.0
Italy	12	0.5	12	0	0.0
Norway	12	0.5	8	4	33.3
Switzerland	12	0.5	6	6	50.0
Finland	11	0.4	9	2	18.2

^1^ SCP—single country paper. ^2^ MCP—multiple country paper.

**Table 2:** List of 25 most productive institutions.

Institution	Ranking ^1^	Country	Articles
University of California	7	USA	155
University of Louisville	NN ^2^	USA	140
University of Toronto	13	CAN	96
University of Gondar	NN	ETH	94
Monash University	34	AUS	78
The University of Newcastle	93	GBR	78
Deakin University	261	AUS	73
University of Washington	21	USA	73
Columbia University	23	USA	66
London School of Hygiene and Tropical Medicine	36	GBR	63
Johns Hopkins University	3	USA	61
Haramaya University	NN	ETH	58
McMaster University	59	CAN	52
Hunter Medical Research Institute	NN	AUS	51
Simon Fraser University	NN	CAN	51
Emory University	64	USA	50
University of British Columbia	25	CAN	49
King’s College London	11	USA	47
University of New South Wales	54	AUS	47
University of Cape Town	102	ZAF	45
University College London	9	GBR	41
University of Manitoba	247	CAN	39
University of Newcastle	NN	GBR	39
University of Pittsburgh	91	USA	39
University of Victoria	NN	AUS	39

^1^ Ranked by 2025 QS World University Rankings for Life Sciences & Medicine. ^2^ Information is unknown.

## Data Availability

The original contributions presented in this study are included in the article. Further inquiries can be directed to the corresponding author.

## Abbreviations

The following abbreviations are used in this manuscript:

USA	United States of America
UK	United Kingdom
ESPAD	European School Survey Project on Alcohol and Other Drugs
MCA	Multiple Correspondence Analysis
